# *Leishmania* DNA detection and species characterization within phlebotomines (Diptera: Psychodidae) from a peridomicile-forest gradient in an Amazonian/Guianan bordering area

**DOI:** 10.1371/journal.pone.0219626

**Published:** 2019-07-15

**Authors:** Thiago Vasconcelos dos Santos, Daniela de Pita-Pereira, Thais Araújo-Pereira, Constança Britto, Fernando Tobias Silveira, Marinete Marins Póvoa, Elizabeth Ferreira Rangel

**Affiliations:** 1 Programa de Pós Graduação em Biologia de Agentes Infecciosos e Parasitários, Instituto de Ciências Biológicas, Universidade Federal do Pará, Belém, Pará State, Brazil; 2 Seção de Parasitologia, Instituto Evandro Chagas, Secretaria de Vigilância em Saúde, Ministério da Saúde, Ananindeua, Pará State, Brazil; 3 Laboratório de Biologia Molecular e Doenças Endêmicas, Fundação Oswaldo Cruz, Instituto Oswaldo Cruz, Rio de Janeiro, Rio de Janeiro State, Brazil; 4 Laboratório Interdisciplinar de Vigilância Entomológica em Diptera e Hemiptera/ Laboratório de Referência Nacional e Internacional/Regional OPAS/OMS de Vigilância Entomológica, Taxonomia e Ecologia de Vetores de Leishmanioses/ Instituto Oswaldo Cruz, Fundação Oswaldo Cruz, Rio de Janeiro, Rio de Janeiro State, Brazil; Academic Medical Centre, NETHERLANDS

## Abstract

In the border region between Brazil and French Guiana, American cutaneous leishmaniasis is a worrisome public health issue, and entomological studies are required there to better identify classical and putative emerging transmission patterns. The present study aimed to detect and characterize *Leishmania* DNA in the phlebotomine population of Oiapoque (Amapá State, Brazil). Phlebotomines were captured in anthropized and wild environments in the outskirts of Oiapoque municipality, using CDC light traps installed in vertical (ground/canopy level) and horizontal (peridomicile/extradomicile/forest-edge/forest) strata. Captured specimens were identified according to their morphology. Females were processed for *Leishmania* DNA detection and characterization using a multiplex polymerase chain reaction targeting kinetoplast DNA (kDNA) and the phlebotomine cacophony gene. The kDNA positive samples were characterized by cloning and sequencing the *Leishmania* 234 bp-*hsp70* gene. Among the 3957 phlebotomine specimens captured, 26 pooled female samples were positive for *Leishmania* (*Viannia*) spp. DNA. Sequencing analysis allowed species-specific identification of *L*. (*V*.) *braziliensis* DNA in *Trichophoromyia ininii*, *Bichromomyia flaviscutellata*, *Nyssomyia umbratilis*, and *Evandromyia infraspinosa*, and *L*. (*V*.) *guyanensis* DNA in *Ny*. *umbratilis*. A pooled sample of *Ny*. *umbratilis* was positive for both *L*. (*V*.) *braziliensis* and *L*. (*V*.) *guyanensis* DNA. The present study provided additional information regarding ACL ecology in Oiapoque, highlighting the presence of *L*. (*V*.) *braziliensis* DNA in different phlebotomine species. The epidemiological implications of these findings and the determinant incrimination of *L*. (*V*.) *braziliensis* as proven vectors in that region must be clarified. In this regard, studies on *Leishmania* spp. infection and suggestive anthropophilic behavior of associated phlebotomines need to be prioritized in entomological surveillance.

## Introduction

Phlebotomine sand flies (Diptera: Psychodidae) are insects of great medical importance owing to their capacity to transmit disease agents, such as *Leishmania* (Kinetoplastida: Trypanosomatidae) parasites, the causative agents of American cutaneous leishmaniasis (ACL)[1–3]. In nature, ACL causative agents are maintained in a complex series of transmission cycles involving a variety of vectors and reservoirs [4]. The impact of natural, ecological, or man-made pressures can further complicate these relationships and lead to the formation of new transmission cycles [5,6].

ACL is endemic to the Amazonian/Guianan region, where it is caused by five parasite species, namely: *Leishmania* (*Viannia*) *guyanensis*, *L*. (*V*.) *braziliensis*, *L*. (*Leishmania*) *amazonensis*, *L*. (*V*.) *lainsoni*, and *L*. (*V*.) *naiffi* [7]. Although *L*. (*V*.) *guyanensis* is considered to be the most important causative agent, potential emerging patterns of infection are worth investigation. For example, in the Oyapock basin, a natural border between the Brazilian state of Amapá and the Ultramarine Department of French Guiana, *L*. (*V*.) *braziliensis* is considered an emerging cause of ACL; reported to be linked to forest encroachment associated with the gold-mining industry [8]. Epidemiological data on the clinical profile of ACL patients in the Brazilian region revealed sporadic mucosal commitment presumed to be caused by this parasite species, drawing attention to a worrisome public health issue [9].

We recently studied ACL ecology in this region, providing comprehensive insights based exclusively on the investigation of the wild environment [6]. However, thelack of data regarding ACL in anthropized environmentsmade usseek further information regarding horizontal stratification of the phlebotomine fauna associated with human dwellings. Therefore, present study aimed to fill some gaps about the eco-epidemiological knowledge of that bordering area, supplying our previous "dissection-based" data on natural infection with those of *Leishmania* DNA detection.

## Materials and methods

### Study area

The municipality of Oiapoque (03°49'29"N, 51°49'05"W) is the most important Brazilian socioeconomic link with the bordering Ultramarine Department of French Guiana. ACL is endemic in this region and is regarded as a “bi-national” issue, asapproximately half of cases appear to be acquired on the Brazilian side, and half on the French Guianan side [9]. Two environments were selected to be surveyed, as follows:

1. Wild environment: Vila Vitória Road (03° 51’ 28.1” N, 51° 48’ 41.3” W), as described in our previous survey [6], a recently opened road that provides eastern access from Oiapoque to Vila Vitória. The forested area shows minimal evidence of human activity and is therefore considered to be well preserved.
2. Anthropized environment: Vila Vitória settlement (3°52'50.8"N, 51°47'35.7"W), a rural settlement surrounded by forest in the Brazilian border region of the Oiapoque River and approximately 6 km from the urban area of Oiapoque. Here, a randomly selected domicile within an environment with known ACL cases was surveyed, taking into consideration to be at the pattern distance (200 to 500m) of the forested area, which is a buffer radius assumed for the preventive measures for ACL transmission by *L*. (*V*.) *guyanensis*, attributed to the apparently limited flight range of the vector *Nysomyia umbratilis*[10–13].

**Fig 1:**
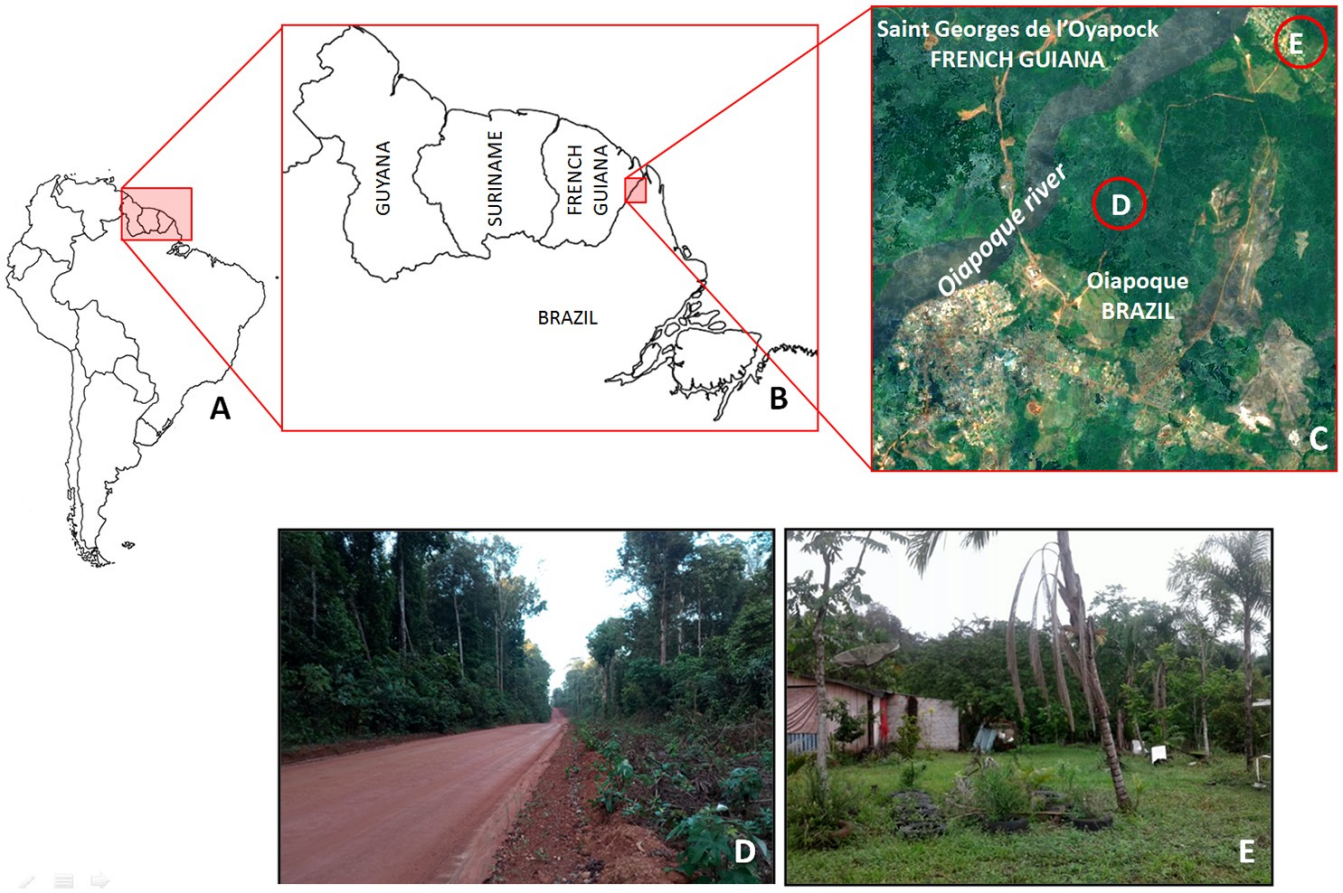
Study area. Located in Guianan Ecoregion Complex, South America (A), the border area between Brazil and French Guiana (B) is socioeconomically linked by the Brazilian municipality of Oiapoque and the French Guianan comune of Saint Georges de l'Oyapock (C), where ACL seems to be occurring in both wild (D) and anthropized (E) environments.

The Fig 1 shows the study area, located in Guianan Ecoregion Complex, South America (A), in the border area between Brazil and French Guiana (B), where in the outskirts of the Brazilian municipality of Oiapoque it can be observed two different environments, wild (D) and anthropized (E), supposed to be ACL *foci*.

### Sampling

Site I was sampled using four CDC light traps installed approximately 100 m in-forest. Two were placed 20 m apart, 1.5m above ground (ground level) and two 20m apart, 20 m above ground (tree canopy level). Captures were performed from November 2016 to October 2017, four nights per month, totalizing 1152 h of sampling (12 h of operation for each CDC x two CDCs *per* site x four nights x 12 months) for each site.

Site II was sampled using four CDC light traps installed 1.5m above ground in a horizontal transect (from the direction of residence to forest), with the following vertical strata: peridomicile, extradomicile, forest-edge, and forest environments. Captures were performed from February to October 2016, four nights per month, totalizing 432 h of sampling (12 h of operation for each CDC x four nights *per* month x nine months) for each site.

Phlebotomines were immediately processed in the field laboratory and stored in 70% ethanol. They were identified using morphological characteristics in fresh conditions or processed for mounting on glass slides using Berlese fluid, according to Ryan [14]. Mounting of females was performed using only the last abdominal segments and head, as the thorax and abdomen were required for *Leishmania* DNA detection and characterization. Taxonomic criteria and nomenclature were adopted following those outlined by Galati et al. [15] and Galati [16].

### *Leishmania* DNA detection and characterization

Procedures for DNA detection and characterization of *Leishmania* species from non-blood-fed phlebotomine females (stored in 70% ethanol) were based on the methodology proposed by Pita-Pereira et al. [17] and Araújo-Pereira et al. [18].

First, a total extract of the macerates of phlebotomine females of the same species without visible blood meal, individually processed or grouped in pools up to 20 specimens, and phlebotomine males (negative controls) were processed for DNA extraction. DNA was extracted using the commercial Wizard SV Genomic DNA Purification System kit (Promega, Madison, USA) according to the manufacturer's specifications. Hot-start multiplex PCR was performed using two pairs of oligonucleotides as follows: The first pair targets a 120bp fragment of the constant region of the kinetoplast DNA (kDNA) mini-circle [oligonucleotide A (5'-GGC CCA TAC ACC AAC CCC-3') and oligonucleotide B (5'-GGG GTA GGG GCG TTC TGC GAA-3')] [19]. The second pair targets the IVS6 region of the phlebotomine cacophony gene [5Lccac (5'-GTG GCC GAA CAT AAT GTT AG-3') and 3Llcac (5'-CCA CGA ACA AGT TCA ACA TC-3')][20]. The inclusion of the second pair of oligonucleotides confers reliability of DNA extraction from phlebotomine samples. The amplified products wereresolved using 2% agarose gel electrophoresis and visualized by staining with Nancy-520 (Merck).

To optimize detection sensitivity, the amplification products were additionally subjected to dot-blot hybridization using a biotinylated probe specific for *Leishmania* (*Viannia*) (5’-TAA TTG TGC ACG GGG AGG CCA-3') [21]. The hybridization reaction was developed using Luminol reagent (Santa Cruz Biotechnology, CA, USA). Positive controls consisted of a pooled sample of experimentally infected *Lutzomyia longipalpis* females, after 72h of feeding on rabbit blood containing 2 × 10^5^ *L*. *braziliensis* parasites/mL.

The DNA recovered from each *Leishmania*-positive sample was subjected to a second, semi-nested-PCR assay targeting the *hsp70* gene. This gene region validated for distinguishing different species of *Leishmania* present in Brazil, with broad coverage of the *L*. (*Viannia*) subgenus. In the first PCR step, a 234 bp fragment of *hsp70* was amplified using the oligonucleotides 5'-GGA CGA GAT CGA GCG CAT GGT-3' and 5'-TCC TTC GAC GCC TCC TGG TTG-3' [22]. In the second step, the same forward oligonucleotide is paired with the following reverse oligonucleotide: 5'-GGA GAA CTA CGC GTA CTC GAT GAA G-3' [23] to amplify a 144 bp internal region of the 234bp fragment. The amplified fragments were purified and cloned into competent *Escherichia coli* DH5α cells using the pGEM T-Easy Vector kit vector (Promega), according to the manufacturer's recommendations.

Sanger sequencing was performed with the RPT01A-PDTIS, Fiocruz-RJ sequencing platform (ABI 3730XL Applied Biosystem) [24], using the BigDye Terminator v3.1 Cycle Sequencing Ready Reaction kit (Applied Biosystems, CA, USA). The electropherograms were initially analyzed using the Phred program [25], and regions with good sequence resolution were submitted for assembly using the CAP3 program [26], Vector sequence removal was performed using the NCBI VecScreen program (http://www.ncbi.nlm.nih.gov/VecScreen/VecScreen.html). Sequences were compared to those available in the BLASTnucleotide database (http://blast.ncbi.nlm.nih.gov/Blast.cgi) using the BLASTN algorithm.

### Data analysis

The Shannon-Wiener (H) diversity index was estimated and evaluated with a *t*-test to compare diversities between the different sites sampled with equal effort (i.e.: wild environment: 1,152 h for each vertical stratum; anthropized environment: 432 h for each horizontal stratum), using the Past software version 3.22 (Øyvind Hammer, Oslo, Norway),[27]. Significance level was set at 5%.

### Environmental issues

Capturing and processing invertebrate fauna (phlebotomines) were authorized by the Sistema de Autorização e Informação em Biodiversidade—SISBIO (Biodiversity Authorization and Information System), under protocol No. 44524.

## Results

**Fig 2:**
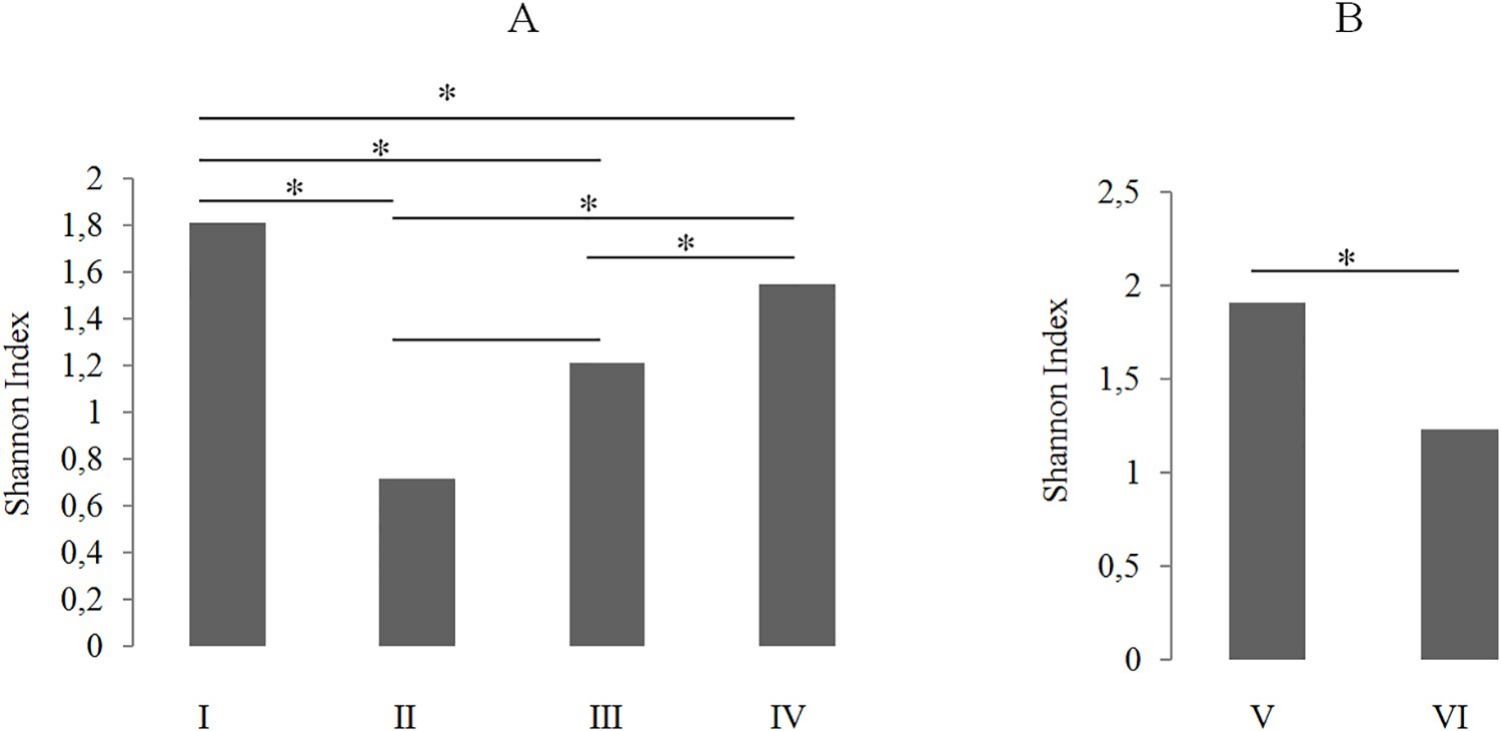
Diversity indexes estimated for the horizontal strata of anthropized environment (A) and for the vertical strata of the wild environment (B). I: peridomicile; II: extradomicile; III: forest edge; IV: forest; V: ground level; VI: canopy level; (*): p< 0.05.

A total of 3957 phlebotomine specimens were captured, 1189 from the anthropized environment and 2768 from the wild environment. Thirty-one species were identified, including *Evandromyia infraspinosa*, *Nyssomyia umbratilis*, and *Trichophoromyia ininii* which were the most frequent, accounting for 2837 (71.7%) of the total number captured. Greatest diversity was observed, for the wild environment, in the ground level (*H* = 1.907) and, for the anthropized environment, in the peridomicile (*H* = 1.809) (Table 1). When comparing diversities within the anthropized environment, all horizontal strata, with exception of extradomicile x forest edge, were significantly different. Within the wild environment, both vertical strata (ground and canopy level) have diversity indexes significantly different (Fig 2).

**Table 1:** Phlebotomine species composition and vertical/horizontal stratification in anthropized and wild environments of Oiapoque, Amapá, Brazil, bordering French Guiana.

Species	Environment						Total	%
	Anthropized				Wild			
	I	II	III	IV	V	VI		
*Evandromyia infraspinosa*	4	2	31	397	716	44	1194	30.2
*Nyssomyia umbratilis*	-	2	7	15	203	899	1126	28.5
*Trichophoromyia ininii*	5	27	249	141	90	5	517	13.1
*Psychodopygus ayrozai*	-	-	-	1	133	148	282	7.1
*Trichopygomyia trichopyga*	-	1	34	136	32	17	220	5.6
*Bichromomyia flaviscutellata*	3	-	1	40	109	7	160	4.0
*Sciopemyia sordellii*	3	-	9	30	76	-	118	3.0
*Psychodopygus squamiventris maripaensis*	-	-	2	8	18	53	81	2.0
*Viannamyia furcata*	2	1	-	5	18	12	38	1.0
*Micropygomyia rorotaensis*	-	-	1	1	18	6	26	0.7
*Evandromyia* sp. of Baduel	1	-	-	-	13	10	24	0.6
*Sciopemyia fluviatilis*	1	-	2	1	17	3	24	0.6
*Psychodopygus claustrei*	-	-	-	-	17	5	22	0.6
*Evandromyia williamsi*	-	-	-	6	12	1	19	0.5
*Evandromyia monstruosa*	-	-	-	6	9	1	16	0.4
*Psychodopygus davisi*	-	-	-	2	3	8	13	0.3
*Psychodopygus geniculatus*	-	-	-	-	4	7	11	0.3
*Psathyromyia aragaoi*	-	-	1	-	3	7	11	0.3
*Viannamyia tuberculata*	-	-	1	5	2	3	11	0.3
*Psychodopygus paraensis*	-	-	-	1	1	6	8	0.2
*Psychodopygus hirsutus*	-	-	-	-	5	2	7	0.2
*Psathyromyia bigeniculata*	-	-	-	-	3	2	5	0.1
*Nyssomyia anduzei*	-	-	-	-	-	5	5	0.1
*Nyssomyia pajoti*	-	-	2	-	1	1	4	0.1
*Psathyromyia dendrophyla*	-	-	-	-	-	3	3	0.1
*Psathyromyia inflata*	-	-	1	-	2	-	3	0.1
*Brumpromyia pintoi*	-	-	-	-	1	1	2	0.1
*Migonemyia migonei*	-	-	-	-	-	2	2	0.1
*Pressatia choti*	-	-	-	-	-	2	2	0.1
*Micropygomyia* (Pilosa Series)	-	-	-	1	1	-	2	0.1
*Psathyromyia abonnenci*	-	-	-	-	-	1	1	0.0
**Total**	**19**	**33**	**341**	**796**	**1507**	**1261**	**3957**	**100**
*Taxa (S)*	7	5	13	17	26	28	31	-
*Diversity (H)*	1.809	0.715	1.029	1.545	1.907	1.231	2.008	-

Sampling effort on the anthropized environment: 432 h for each stratum; Sampling effort on the wild environment: 1152 h for each stratum; I: peridomicile; II: extradomicile; III: forest edge; IV: forest; V: ground level; VI: canopy level;

(*): p< 0.05.

Phlebotomine DNA extraction was considered successful in 551 samples that amplified the cacophony gene; and thus appropriate for *Leishmania spp*. DNA detection. Engorged phlebotomines were excluded from the infection assays due to the risk of detecting a non-established infection in flies that had recently consumed blood from an infected source.

Twenty-six samples were positive for *Leishmania* (*Viannia*) spp. DNA. In the anthropized environment, *Leishmania* (*Viannia*) spp. DNA was detected in forest-edge samples of *Psychodopygus squamiventris maripaensis* (n = 1) and *Bichromomyia flaviscutellata* (n = 3), and in forest samples of *N*. *umbratilis* (n = 1) and *Th*. *ininii* (n = 2). In the wild environment, *Leishmania* (*Viannia*) spp. DNA was detected in *B*. *flaviscutellata* (n = 1) and *E*. *infraspinosa* (n = 2) caught at ground level, in *N*. *umbratilis* from ground (n = 4) and canopy (n = 9) levels, and in *P*. *s*. *maripaensis* from ground (n = 1) and canopy (n = 2) levels.

The minimum rate of DNA detection (number of positive samples / total samples tested x 100) was calculated for each the five species captured and positive for *Leishmania* spp. DNA: *P*. *s*. *maripaensis* (18.1%), *N*. *umbratilis* (5.8%), *Bi*. *flaviscutellata* (7%), *E*. *infraspinosa* (2.8%) and *T*. *ininii* (1.2%). *Leishmania* species identification was successful in 8 of the 26 positive samples, with coverage varying from 80–97% and identity from 88–100%. It was not possible to characterize the others due to cloning failures resulting from low DNA levels or incompatibility with the available sequences in the database.

Sequencing analyses are summarized in Table 2. BLAST analysis with sequences available from GenBank revealed the presence of *L*. (*V*.) *braziliensis* DNA in one *T*. *ininii* (10 specimens) sample from the forest-edge, one of *B*. *flaviscutellata* (6 specimens) from the forest, two of *N*. *umbratilis* (10 specimens, each) from canopy level, and two of *E*. *infraspinosa* (10 specimens, each) from ground level. Two samples of *N*. *umbratilis* (10 specimens each) from the canopy level were positive for *Leishmania* DNA, one was identified as *L*. (*V*.) *guyanensis* and the other contained DNA from both *L*. (*V*.) *braziliensis* and *L*. (*V*.) *guyanensis*.

**Table 2:** Samples of phlebotomines PCR-multiplex-positive for the cacophony gene (Phlebotominae) and for the kDNA gene (*Leishmania* spp.) with descriptions of the environment where they were captured, number of specimens analyzed and sequencing results.

	IOC code	Phlebotomine species	Environment		N	*Leishmania* species (*hsp*70)	Blast analysis		
			Anthropized	Wild			Cover (%)	Identity (%)	Access number
1	5/16.4.mar.D26	*Psychodopygus squamiventris maripaensis*	forest	-	6	NI	-	-	-
2	5/16.4.fla.D27	*Bichromomyia flaviscutellata*	forest	-	10	NI	-	-	-
3	5/16.4.fla.D28	*Bichromomyia flaviscutellata*	forest	-	5	NI	-	-	-
4	5/16.co.umb.D30	*Nyssomyia umbratilis*	-	CDC canopy	10	NI	-	-	-
5	5/16.co.umb.D33	*Nyssomyia umbratilis*	-	CDC ground	10	NI	-	-	-
6	5/16.so.umb.D35	*Nyssomyia umbratilis*	-	CDC ground	3	NI	-	-	-
7	6/16.3.umb.D37	*Nyssomyia umbratilis*	border	-	5	NI	-	-	-
8	6/16.3.ini.D38	*Trichophoromyia ininii*	border	-	10	*L*. (*V*.) *braziliensis*	96	98	GU368187.1
9	6/16.3.ini.D39	*Trichophoromyia ininii*	border	-	10	NI	-	-	-
10	6/16.4.fla.D41	*Bichromomyia flaviscutellata*	forest	-	6	*L*. (*V*.) *braziliensis*	94	100	GU368187.1
11	6/16.co.umb.D46	*Nyssomyia umbratilis*	-	CDC canopy	10	NI	-	-	-
12	8/16.co.mar.D53	*Psychodopygus squamiventris maripaensis*	-	CDC canopy	2	NI	-	-	-
13	8/16.co.umb.D54	*Nyssomyia umbratilis*	-	CDC canopy	10	*L*. (*V*.) *braziliensis*	96	89	XM_001566273
14	8/16.co.umb.D55	*Nyssomyia umbratilis*	-	CDC canopy	10	*L*. (*V*.) *braziliensis*	91	89	XM_001566273
15*	8/16.co.umb.D56	*Nyssomyia umbratilis*	-	CDC canopy	10	*L*. (*V*.) *guyanensis/ L*. (*V*.) *braziliensis*	89/97	94/99	HF586362.1/ XM_001566273
16	8/16.co.umb.D57	*Nyssomyia umbratilis*	-	CDC canopy	10	NI	-	-	-
17	8/16.co.umb.D58	*Nyssomyia umbratilis*	-	CDC canopy	10	*L*. (*V*.) *guyanensis*	97	100	HF586362.1
18	9/16.co.umb.D60	*Nyssomyia umbratilis*	-	CDC canopy	10	NI	-	-	-
19	9/16.co.umb.D61	*Nyssomyia umbratilis*	-	CDC canopy	5	NI	-	-	-
20	9/16.so.mar.D62	*Psychodopygus squamiventris maripaensis*	-	CDC ground	1	NI	-	-	-
21	9/16.so.fla.D63	*Bichromomyia flaviscutellata*	-	CDCground	4	NI	-	-	-
22	9/16.so.umb.D64	*Nyssomyia umbratilis*	-	CDC ground	10	NI	-	-	-
23	9/16.so.umb.D65	*Nyssomyia umbratilis*	-	CDC ground	10	NI	-	-	-
24	1/17.co.mar.D69	*Psychodopygus squamiventris maripaensis*	-	CDC canopy	3	NI	-	-	-
25	1/2.so.inf.D74	*Evandromyia infraspinosa*	-	CDC ground	10	*L*. (*V*.) *braziliensis*	95	88	XM_001566273.2
26	1/2.so.inf.D75	*Evandromyia infraspinosa*	-	CDC ground	10	*L*. (*V*.) *braziliensis*	80	89	XM_001566273.2

*: duplicate sample, since it generated clones of two different species of *Leishmania*;

N: number of specimens in the sample, ranging from individual samples to pools of up to ten specimens; NI: not identified at species level by sequencing. Minimal detection rate for positive phlebotomine species: *Psychodopygussquamiventrismaripaensis*(18.1%), *Nyssomyiaumbratilis*(5.8%), *Bichromomyiaflaviscutellata* (7%), *Evandromyiainfraspinosa*(2.8%) and *Trichophoromyiaininii* (1.2%).

## Discussion

In Brazil, ACL presents three characteristic epidemiological patterns: 1) Sylvatic, where transmission occurs mainly in primary forest environments; considered a zoonosis of wild animals with occasional human cases in areas of recent colonization due to the penetration of the man into the wild. 2) Occupational/recreational, associated with disordered forest exploration and with the clearing of forests for the construction of roads, hydroelectric power plants, village settlements, timber extraction, agricultural and military training activities, and ecotourism. 3) Rural and peri-urban in colonization areas, related to the migratory process, occupation of slopes and clusters in urban centers associated with secondary or residual forests [13]. Of these, the two former patterns are well recognized in the Amazon region, and support the hypothesis to be occurring in the studied area.

As hypothesized, the phlebotomine species composition observed in this study were highly similar to that recently presented in our previous study [6]. Therefore, to avoid redundancy, the present discussion here is focused on the results of horizontal stratification and *Leishmania* DNA detection within the phlebotomine population.

In the wild environment, diversity was significantly higher in the ground (*H* = 1.907; p < 0.05), where CDCs are usually set spatially congruent with the flight level of majority of phlebotomine species. On the other hand, canopy stratum comprise a ecological subsystem with fewer number of species with close relationship with arboreal vertebrates, such as occur with *N*. *umbratilis* and sloths and birds [6]. In the anthropized environment, differences on the diversity indexes were statistically significant when comparing between each stratum, with exception of extradomicile x forest edge. Similar environmental pressure and ecological niche between these sampling points may explain this apparent similarity. Greatest diversity found in the peridomicile (*H* = 1.809; p < 0.05) can be attributed to the search for shelter and offer of blood of domestic animals [13, 19].

Eight phlebotomine species were found in the extra-forest environments (peridomicile/extradomicile strata). Among these, it is important to highlight *B*. *flaviscutellata*, this species can be found in primary and secondary forests [28], and may be progressively adapting to environments modified by man [29,30]. Several studies have demonstrated the potential of peridomiciliarization of this species in the Amazonian region [31–34]; however, the peridomiciliary populations were usually small, compared to those in the forest stratum. The higher frequency of *Bi*. *flaviscutellata* in a forested environment, compared to the peridomiciliary ecotope is consistent with the findings of other studies [30, 35–37]. Therefore, there is no concrete evidence herethat this species has established peridomiciliary colonization. This assertion is further supported by a study conducted within the Bragança region of Pará, where pregnant *Bi*. *flaviscutellata* females were found only in the forest environment [38].

Molecular tools have been gradually replacing the traditional technique of phlebotomine dissection and provide several advantages, particularly with respect to sensitivity and specificity [39] allowing the detection of a single parasite [17]. However, it cannot be denied that parasite isolation significantly extends the range of investigations which can be performed. Therefore, it is suggested that, in entomological studies, DNA detection techniques and molecular characterization of *Leishmania* spp. be combined with dissection and attempted parasite isolation. Present results support our previous “dissection-based” information about *Leishmania* parasites in the Oiapoque environment [6].

The minimum detection rate observed by the molecular method (4.7%) was higher than that demonstrated in our early experience in Oiapoque using the dissection method (0.78%) [6], which is apparently attributed to the recognized high sensitivity of PCR and its variants [17,39]. However, we recognize that, in hands of experienced team and in females without a visible bloodmeal, the microscopical examination of dissected midguts is equally sensitive as Q-PCR, as has been proven elsewhere [40]. In addition, there are several other possible explanations to these differences of infection/detection rates, like the effect of season, micro-location of trapping sites, that should not be neglected in such comparisons.

It is important to note that these values do not necessarily reflect the risk of human infection; other factors should be considered in assessing the risk of exposure to ACL agents, such as the degree of anthropophilia of their potential vectors [3].

The cloning and sequencing stages are important because phlebotomines present a complex biological material and although kDNA detection provides high sensitivity, it does not allow identification to species level. Using this method, identification is possible at the level of genus and subgenus and this is a particular problem with the subgenus *Viannia*. Unfortunately, the best targets for genotyping, such as *Hsp*70, are not good detection targets due to low copy number. Therefore, effective detection and identification of *Leishmania* spp. from phlebotomines requires a primary detection step targeting kDNA, followed by a subsequent genotyping step using another molecular target, such as *hsp70*. Even so, the efficiency is reduced, due to the low amount of target DNA, and then we consider some identity values lower than 90%. Although we were unable to characterize 18 of the 26 positive samples, the sequence of eight pools showed compatible coverage and identity to contemplate characterization at species-specific level; providing important information in the context of Amazonian/Guianan ACL.

*L*. (*V*.) *guyanensis* DNA was detected in several samples of *N*. *umbratilis*, supporting the whole context that this natural vector x parasite relationship that has been discussed in the course of our past experience in Oiapoque [6] and based on the extensive literature from the Amazon region [3,4,10,41–45]

*L*. (*V*.) *braziliensis* DNA was detected in four different phlebotomine species: *T*. *ininii*, *B*. *flaviscutellata*, *E*. *infraspinosa*, and *N*. *umbratilis*. In Brazil, *L*. (*V*.) *braziliensis* transmission is associated with the highest number of known vectors, where there are 17 phlebotomine species with proven or potential links to its transmission; seven of which are only associated by molecular findings [4]. The wide geographical distribution and genetic diversity of *L*. (*V*.) *braziliensis* strains [46] may contribute, in part, to the large list of its vectors. Furthermore, these transmission cycles may be very specific, as each leishmanian ecotope is reported to be spatiotemporally unique [6].

Interestingly, *L*. (*V*.) *braziliensis* DNA has not been found previously in any of these four phlebotomine species. Conversely, *B*. *flaviscutellata* and *N*. *umbratilis* play well-recognized roles in the transmission of the respectively associated parasites, *L*. (*L*.) *amazonensis* and *L*. (*V*.) *guyanensis* [2,4]. *Leishmania* sp. DNA was detected in *Trichophoromyia ininii*, considered a low-anthropophilic phlebotomine species, in Sabajo Heuvels, Suriname [47]. *E*. *infraspinosa* had previously been found in Oiapoque carrying promastigotes that were subsequently characterized as *L*. (*V*.) *guyanensis* from the residual material present on the slide [6].

The detection of *L*. (*V*.) *braziliensis* in four different phlebotomine species suggests concomitant contact of these insects with potential reservoirs of the parasite. Considering that rodents are the most likely animals to be involved in maintaining the enzootic cycle of *L*. (*V*.) *braziliensis* [48–51], it is rationally inferential that these phlebotomines are feeding and ingesting parasitic forms in a competent source of infection within a compatible spatiotemporal context. The potential feeding habits of three, of these four species of sand fly, is a corroborating factor for this statement. Rodentophilia is undoubtedly well recognized for *B*. *flaviscutellata* [52] and *N*. *umbratilis* has been found alternatively feeding on blood from rodents in an environmentally-impacted area close to Manaus, Amazonian Brazil [53]. Additionally, *E*. *infraspinosa*, which infrequently bites man in the bordering region of Jari, between the Brazilian states of Pará and Amapá, was relatively frequent species in Disney-trapped rodent trap catches [10]. Furthermore, *Dasyprocta leporina* DNA was recently detected in *E*. *infraspinosa* in Saint Georges de l'Oyapock, Guiana [54]. Still in regard of *E*. *infraspinosa*, in our past experience during captures in area I, this species has drawn medical attention for having been observed biting professionals during Shannon trap captures in same circumstances of having been found naturally infected by *L*. (*V*.) *guyanensis* [6]. Conversely, other populations of this fly species may present distinct feeding behavior. For example, previous studies of anuran trypanosomatids carried by this phlebotomine species in the western Amazon led to the hypothesis that it feeds on cold-blooded animals in this ecotope [55]. These findings stress the need for further investigation of *E*. *infraspinosa* with respect to *L*. (*V*.) *braziliensis*-associated ACL transmission; particularly as these phlebotomine populations may also be permissive to other *Leishmania* spp. and have suggestive anthropophilic behavior.

One pooled sample of ten females of *N*. *umbratilis* was concomitantly positive for *L*. (*V*.) *braziliensis* and *L*. (*V*.) *guyanensis* DNA; however, it cannot be ascertained whether these two parasites were within the same phlebotomine or in different specimens of that pool. In this study and in the literature [53–56] it is convergent with the possibility of *N*. *umbratilis* feed on different animals that act as potential reservoirs of *Leishmania* spp.

## Conclusions

The results of the present study provide additional information on ACL ecology in Oiapoque, highlighting the presence *L*. (*V*.) *braziliensis* DNA in different phlebotomine species captured in ecologically distinct environments and strata in the study area. It should be noted, however, that these findings alone are not sufficient to conclude that there is a transmission cycle involving these phlebotomines and *L*. (*V*.) *braziliensis*. First, absence of visible blood cannot be interpreted as the possible survival of the parasite. Second, and more importantly, studies focused to this topic demonstrated that parasite DNA is detectable few days after *Leishmania* are killed in the non-natural vectors [57,58], reinforcing that PCR-based findings, when analyzed in isolation, are not enough for vector incrimination. Considering established natural infection, the epidemiological importance of these potential transmission cycles still require further evidence. Quantification of the parasitic load and/or demonstration of infective forms would certainly present a further step towards improving knowledge of the potential vectors of *L*. (*V*.) *braziliensis* in this region. Nonetheless, the known capacity of these species to harbor *Leishmania* spp. combined with suggestive anthropophilic behavior present a need for prioritized entomological surveillance.
